# Evaluating the Effect of RNA Interference of X-Box Binding Protein 1 (*XBP1*) on Survival and Fecundity in *Acyrthosiphon pisum*

**Published:** 2026-03-16

**Authors:** Jacob Lutgen, James Balthazor

**Affiliations:** 1 Department of Chemistry, Fort Hays State University, Hays, United States of America

**Keywords:** RNAi, *XBP1*, *Acyrthosiphon pisum*, Unfolded protein response, Pest management, dsRNA, Knockdown, Aphid, Survival, Fecundity

## Abstract

*Acyrthosiphon pisum* (pea aphid) is a major pest of Fabaceae (legume) crops, causing direct feeding damage and transmitting plant viruses. Conventional control relies on broad-spectrum insecticides and natural enemies, both of which can harm non-target organisms and ecosystems. RNA interference (RNAi) offers a promising, species-specific alternative by silencing essential genes *via* double-stranded RNA (dsRNA). This study targeted X-Box Binding Protein 1 (*XBP1*), a key transcription factor in the Unfolded Protein Response (UPR) pathway that regulates proper protein folding in the Endoplasmic Reticulum (ER). This chaperone promotes folding of newly synthesized polypeptides and helps manage misfolded proteins. Silencing the *XBP1* gene was hypothesized to cause accumulation of misfolded proteins in the ER lumen, triggering ER stress, UPR overload and ultimately apoptosis, leading to aphid mortality.

Total RNA was extracted from adult pea aphids, reverse-transcribed to cDNA and used to synthesize gene-specific dsRNA for *XBP1*. The dsRNA was conjugated to Branched-Amphiphilic Peptide Capsules (BAPCs) and delivered orally at concentrations of 1, 10 and 100 ng/μL *via* artificial diet feeding in parafilm-layered petri dishes. Fecundity and survival were monitored at regular intervals. Results demonstrated a fifty percent decrease in fecundity and a thirty percent decrease in survival in the 100 ng/μL treatment groups compared to controls (log-rank test), supporting the induction of apoptosis. This approach highlights the potential of UPR-targeted RNAi for eco-friendly, targeted pest control of *A. pisum*.

## INTRODUCTION

*Acyrthosiphon pisum* is an insect that is a significant pest to alfalfa and other legumes. Currently, the main mechanisms used to protect fields against pea aphids are insecticides and early cutting of the field [1]. Making genetically modified plants that can alter protein functions within aphids, leading to death of the aphid, would be the ideal situation to control the aphid population. As soon as aphids begin to feed on the crop, their protein functions would change and they would be killed. Before this is possible, a gene must be identified that kills aphids efficiently to be worth modifying into crops. *A. pisum* are used as model insects because their genome is entirely sequenced and they are easy to sustain in laboratories, growing on fava beans, *Vicia faba* [2].

The X-box Binding Protein 1 (*XBP1*) is a pivotal transcription factor in the Unfolded Protein Response (UPR), an evolutionarily conserved cellular pathway that maintains Endoplasmic Reticulum (ER) homeostasis during stress caused by accumulation of unfolded or misfolded proteins [3,4]. ER stress triggers the UPR through three main branches: Inositol-Requiring Enzyme 1 (IRE1), PKR-like ER Kinase (PERK) and Activating Transcription Factor 6 (ATF6). Among these, the IRE1α-*XBP1* arm is the most conserved and plays a central role in adaptive responses.

Under basal conditions, IRE1α remains inactive, bound to the chaperone BiP/GRP78 in the ER lumen. Upon ER stress, unfolded proteins sequester BiP, leading to IRE1α dimerization/oligomerization, autophosphorylation and activation of its endoribonuclease domain [5,6]. Activated IRE1α then performs unconventional splicing of *XBP1* mRNA by excising a specific 26-nucleotide intron in metazoans (including insects), causing a translational frameshift. This generates the spliced form, *XBP1*s, a potent Basic Leucine Zipper (bZIP) transcription factor. *XBP1*s translocates to the nucleus and induces expression of target genes encoding ER chaperones (e.g., BiP/GRP78, GRP94), folding enzymes (e.g., PDI), lipid biosynthetic enzymes for ER membrane expansion and components of ER-Associated Degradation (ERAD) machinery (e.g., EDEM, Derlin). These changes enhance protein folding capacity, promote ER biogenesis and facilitate clearance of misfolded proteins to restore proteostasis and promote cell survival.

In contrast, if ER stress persists unresolved, the UPR shifts toward pro-apoptotic signaling. Sustained IRE1α activity recruits TRAF2, activating ASK1 and downstream JNK/p38 MAPK pathways that promote apoptosis. Additionally, other UPR branches (e.g., PERK-mediated induction of CHOP) dominate, upregulating pro-apoptotic factors and downregulating anti-apoptotic proteins (e.g., Bcl-2 family members) [7]. Knockdown or silencing of *XBP1* disrupts this adaptive IRE1α-*XBP1* axis, impairing chaperone induction and ERAD, leading to exacerbated accumulation of misfolded proteins in the ER lumen, intensified chronic ER stress and a bias toward pro-apoptotic cascades (including caspase activation), ultimately resulting in cell death *via* apoptosis.

In insects such as the pea aphid *Acyrthosiphon pisum*, which experiences high secretory demands (e.g., for saliva production and virus transmission), the IRE1-*XBP1* pathway is essential for proteostasis under physiological or environmental stress although limited direct studies exist on *XBP1* in aphids, the UPR mechanism is highly conserved across metazoans, including insects. Species-specific RNAi targeting *XBP1* is thus hypothesized to induce chronic ER stress, cellular dysfunction and organismal lethality, offering a targeted pest control strategy with minimal off-target effects on non-pest species, as the pathway’s core components are conserved but sequence-specific dsRNA enables selectivity [8,9]. This study aims to investigate the effects of *XBP1*-dsRNA on the fecundity and survival of adult *Acyrthosiphon pisum*. Species-specific interference of *A. pisum XBP1* would allow for the prevention of pea aphid infestations without the use of insecticides and the possibility of harming other species and the environment [10–14]. Ideally, transgenic legumes could be produced to endogenously express dsRNA to interfere with pea aphid *XBP1*. We hypothesize that RNA interference of the *XBP1* gene by treatment with *XBP1*-dsRNA will result in a decrease in survival and fecundity of *A. pisum* [15–18].

## MATERIALS AND METHODS

### Whole RNA Isolation

Total RNA was isolated from adult pea aphids (*Acyrthosiphon pisum*) using TRIzol reagent. Ten adult aphids were homogenized in a micro centrifuge tube containing 1 mL of TRIzol to lyse cells and fractionate cellular components. The homogenate was centrifuged to pellet debris and separate phases. The supernatant was transferred to a new tube, mixed with chloroform by vortexing and centrifuged to separate the mixture into an upper aqueous layer (containing RNA), an interphase and a lower organic layer. The aqueous layer was carefully transferred to a fresh micro centrifuge tube, mixed with isopropanol by brief vortexing and centrifuged to precipitate the RNA as a pellet. The pellet was washed with 75% ethanol, centrifuged again and allowed to air-dry. Finally, the RNA pellet was dissolved in ultrapure water and its concentration and purity were determined using a nano drop spectrophotometer.

### cDNA Synthesis

First-strand cDNA was synthesized from 1 μg of total RNA using the single shot SYBR Green Kit according to the manufacturer’s protocol. Following synthesis, the cDNA product was verified by agarose gel electrophoresis to confirm successful reverse transcription. The concentration and purity of the cDNA were subsequently quantified using a nano drop spectrophotometer.

### dsRNA Synthesis

Double-stranded RNA (dsRNA) targeting *XBP1* was synthesized from 1 μg of cDNA using the MEGAscript Kit according to the manufacturer’s instructions. Gene-specific primers designed for the *A. pisum XBP1* sequence were incorporated to generate the target dsRNA. The synthesized dsRNA was confirmed by agarose gel electrophoresis and its concentration and purity were measured using a NanoDrop spectrophotometer.

### Preliminary Fecundity Study

To assess the impact on reproduction, 50 adult aphids were placed in a petri dish lined with parafilm to contain them. *XBP1*-dsRNA, conjugated to Branched-Amphiphilic Peptide Capsules (BAPCs), was added to Akey-Beck artificial diet at final concentrations of 100 ng/μL, 10 ng/μL, or 1 ng/μL and applied to the top layer of parafilm. A second layer of parafilm was then spread over the dish, sandwiching the diet between the two layers to allow feeding. The number of aphids (including nymphs as indicators of fecundity) in each petri dish was counted every 12 hours.

### Feeding Studies

For survival assays, 50 adult aphids were introduced into petri dishes prepared as described above. *XBP1*-dsRNA conjugated to BAPCs was incorporated into akey-beck artificial diet at concentrations of 100 ng/μL, 10 ng/μL, 1 ng/μL, or 0 ng/μL (control) and presented between parafilm layers. Mortality of the original adult aphids was monitored by counting deaths every 3 hours until 50 deaths occurred in the cohort. Survival curves were generated and differences between treatment groups and the control were evaluated using the log-rank test.

## RESULTS

**Figure 1:**
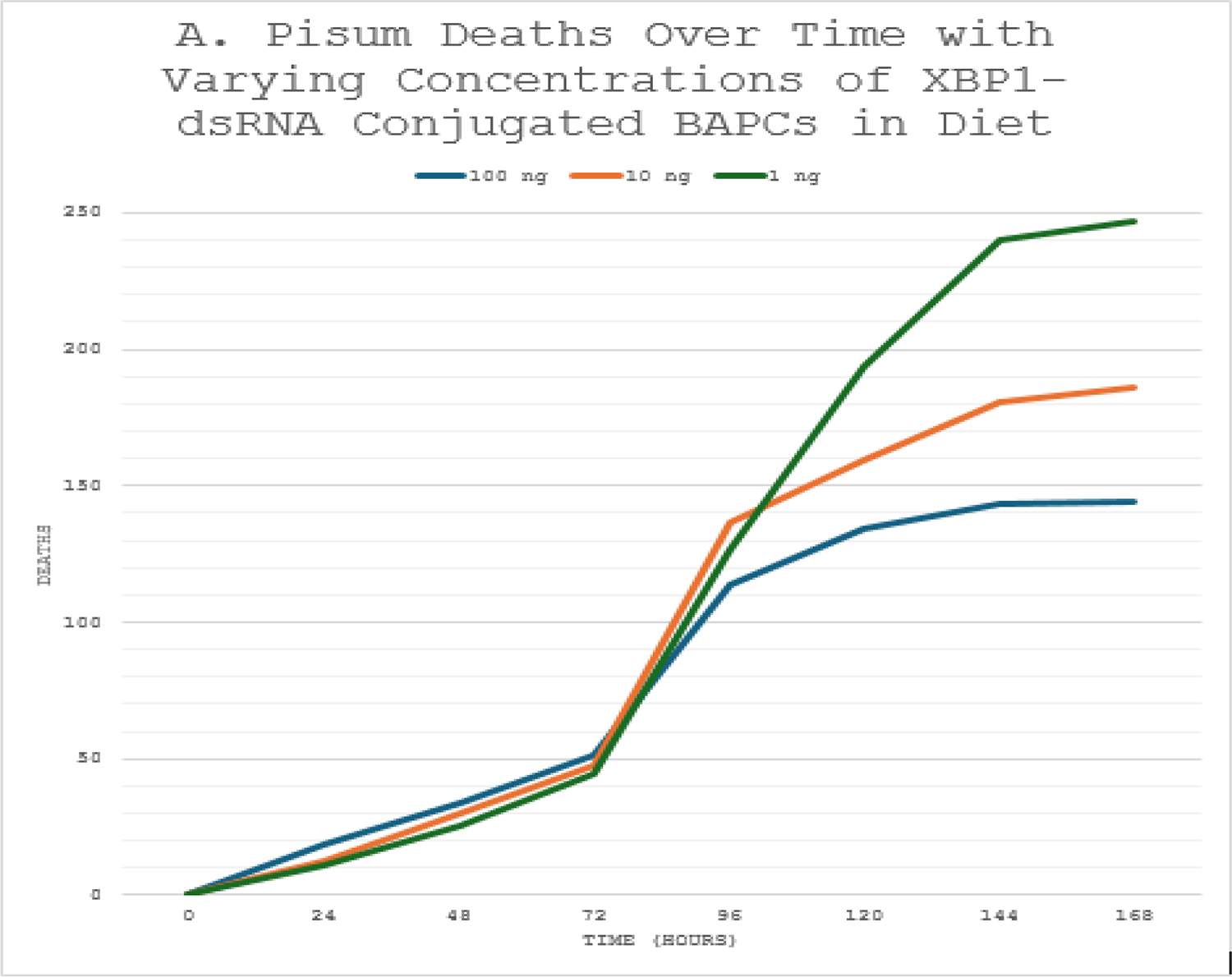
Aphid population over time with varying concentrations of *XBP1*-dsRNA conjugated BAPCs (Fecundity study).

Oral administration of *XBP1*-dsRNA conjugated to Branched-Amphiphilic Peptide Capsules (BAPCs) produced clear dose-dependent effects on both fecundity and survival in adult *Acyrthosiphon pisum*. In the preliminary fecundity assay, aphids fed artificial diet containing varying dsRNA concentrations (1, 10, or 100 ng/μL) showed marked differences in population growth over time, primarily driven by nymph production (Figure 1). The 1 ng/μL group exhibited the highest cumulative aphid numbers, reaching approximately 300 individuals by 168 hours, indicative of robust reproductive output. In contrast, the 100 ng/μL treatment group displayed substantially reduced fecundity, with population levels plateauing near 150 individuals a 50% decrease relative to the lowest-dose group. The 10 ng/μL treatment showed intermediate results, suggesting a threshold effect where only higher dsRNA exposure meaningfully impairs reproduction. These outcomes align with expected disruption of ER proteostasis, as *XBP1* silencing would limit chaperone support needed for high secretory demands during oogenesis and nymph development.

**Figure 2:**
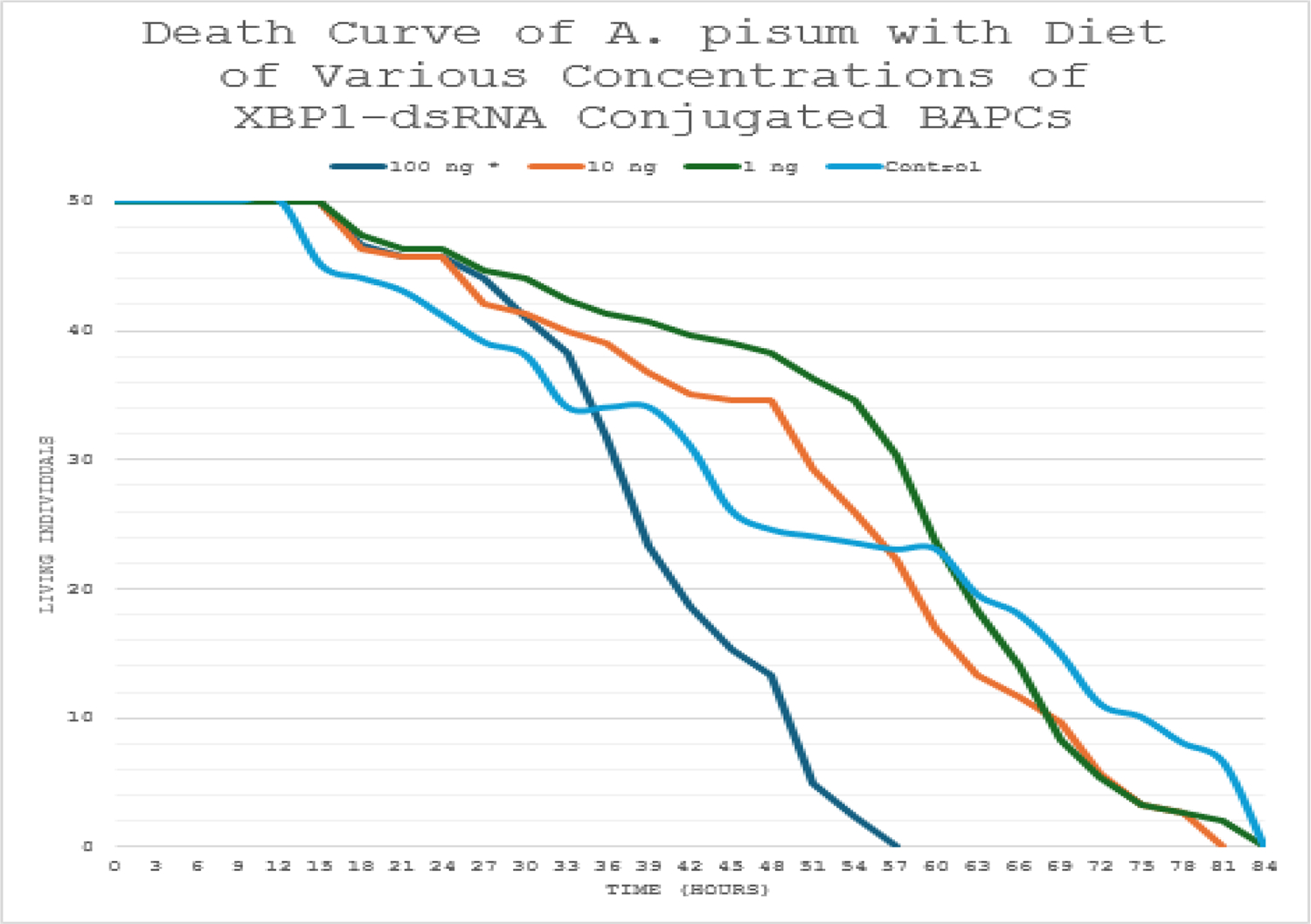
Death curve of *A. pisum* with diet of varying concentrations *XBP1*-dsRNA conjugated BAPCs (Feeding study until death).

Kaplan-Meier survival analysis of aphids under continuous dsRNA exposure (until 50 deaths in each cohort) demonstrated a significant reduction in longevity at the highest dose (Figure 2). Survival curves for the 100 ng/μL group diverged from controls starting around 24–36 hours, with accelerated mortality leading to approximately 30% fewer surviving individuals by the end of monitoring (84 hours). Log-rank testing confirmed statistically significant differences between the 100 ng/μL treatment and the control group (p<0.05). Lower concentrations (1 ng/μL and 10 ng/μL) produced survival curves that closely overlapped the control (p>0.05), indicating no detectable impact at these levels.

**Figure 3:**
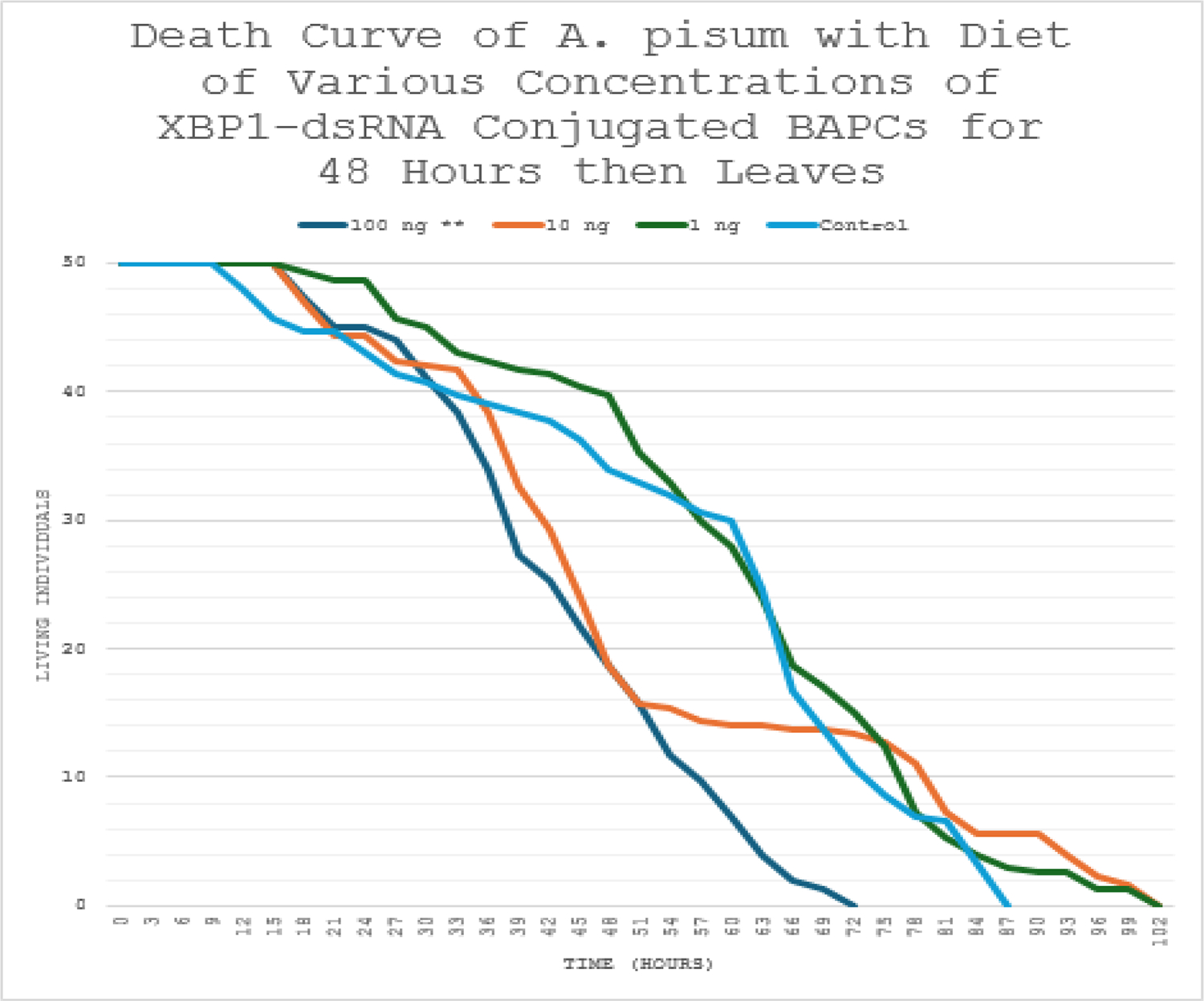
Death curve of *A. pisum* with diet of varying concentrations *XBP1*-dsRNA conjugated BAPCs for 48 hours followed by normal diet.

A parallel feeding study limited dsRNA exposure to 48 hours, followed by a return to untreated diet, yielded comparable results (Figure 3). The 100 ng/μL group again showed earlier and more pronounced declines in living individuals, resulting in a similar 30% overall survival reduction relative to controls. Kaplan-Meier curves separated noticeably within the exposure window (by ~36–48 hours), with persistent differences there - after despite normal diet resumption (log-rank test, p<0.05 *vs*. control). No significant survival differences were observed for the 1 ng/μL or 10 ng/μL groups (p>0.05). This persistent effect suggests that brief high-dose exposure induces irreversible ER stress and apoptotic progression in affected cells.

Overall, only the 100 ng/μL concentration elicited biologically meaningful and statistically supported reductions in both fecundity (50%) and survival (30%), consistent with a concentration-dependent requirement for effective RNAi-mediated *XBP1* silencing in *A. pisum*. Lower doses appeared insufficient to achieve threshold knockdown, as evidenced by survival and reproductive outcomes indistinguishable from untreated controls.

The initial study was to observe the differences in fecundity of *A. pisum* when treated with varying concentrations of *XBP1*-dsRNA conjugated to BAPCs. As expected, the lowest concentration of dsRNA resulted in the lowest number of initial deaths and the greatest fecundity of the three treatments. The 100 ng treatment resulted in a fifty percent decrease in fecundity.

The death curve of the feeding study with various concentrations of *XBP1*-dsRNA conjugated to BAPCs in the diet until death. Statistically significant differences were found between the 100 ng* treatment and the control group using a log-rank test (p<0.05). The 100 ng treatment resulted in a thirty percent decrease in survival.

The death curve of the feeding study with various concentrations of *XBP1*-dsRNA conjugated to BAPCs for 48 hours followed by a normal diet. Statistically significant differences were found between the 100 ng** treatment and the control group using a log-rank test (p<0.05). A similar decrease in survival of thirty percent in the 100 ng treatment is observed.

## DISCUSSION AND CONCLUSION

RNA interference (RNAi) targeting X-Box Binding Protein 1 (*XBP1*) significantly impaired both fecundity and survival in adult *Acyrthosiphon pisum* (pea aphid) under controlled feeding conditions. In the preliminary fecundity assay, oral delivery of *XBP1*-dsRNA conjugated to Branched-Amphiphilic Peptide Capsules (BAPCs) at 100 ng/μL resulted in a 50% reduction in offspring production compared to the 1 ng/μL treatment group, consistent with dose-dependent disruption of reproductive output. This aligns with the role of *XBP1* as a central regulator of the Unfolded Protein Response (UPR) in the Endoplasmic Reticulum (ER), where its silencing is expected to promote accumulation of misfolded proteins, induce ER stress and ultimately trigger apoptosis in affected cells, thereby limiting nymph production.

Survival analyses further supported the efficacy of *XBP1* knockdown. In the continuous feeding study (Figure 2), aphids exposed to 100 ng/μL *XBP1*-dsRNA exhibited a 30% reduction in overall survival compared to the control group, with Kaplan-Meier survival curves diverging markedly over time. Log-rank testing confirmed statistically significant differences between the 100 ng/μL treatment and control (p<0.05), whereas lower concentrations (1 ng/μL and 10 ng/μL) showed no significant deviation from control survival (p>0.05). A similar pattern emerged in the short-term exposure study (Figure 3), where 48-hour feeding of 100 ng/μL *XBP1*-dsRNA followed by a normal diet still produced a comparable 30% decrease in survival, again with significant separation from controls *via* log-rank test (p<0.05) and no effects at lower doses. These results indicate that even brief exposure to high-dose dsRNA elicits persistent lethality, likely due to irreversible ER stress and apoptotic cascades downstream of UPR dysregulation.

The observed threshold effect at 100 ng/μL suggests a concentration-dependent requirement for sufficient dsRNA uptake and processing to achieve meaningful gene silencing in *A. pisum*, consistent with prior reports of dose-sensitive RNAi in aphids using oral delivery systems. The lack of significance at lower doses underscores the importance of optimizing delivery vehicles (such as BAPCs) and exposure regimens for practical pest management applications. Importantly, these effects were specific to the targeted gene, as non-target controls (e.g., ongoing tests in unrelated systems like hela cells) are expected to show no response, supporting species-specificity and reduced off-target risks compared to broad-spectrum insecticides.

While this study demonstrates proof-of-principle for UPR-targeted RNAi as a viable strategy to suppress *A. pisum* populations, several limitations remain. Survival reductions were moderate (30%) and fecundity impacts were assessed indirectly *via* population counts rather than direct quantification of knockdown efficiency. Future work will employ quantitative PCR (qPCR) to measure *XBP1* transcript and protein levels across treatment groups, with the expectation of greater knockdown at higher dsRNA concentrations and minimal changes at lower doses or in controls. Additional studies will explore synergistic effects of combining *XBP1*-dsRNA with other dsRNAs targeting complementary pathways, which may enhance lethality and overcome potential resistance mechanisms. Successful validation of these approaches could pave the way for transgenic legumes engineered to express *A. pisum*-specific dsRNA, offering an environmentally sustainable, pest-specific alternative to current management practices reliant on chemical insecticides or cultural controls.

In summary, RNAi-mediated silencing of *XBP1* significantly reduces survival (log-rank p<0.05 at 100 ng/μL) and fecundity in *A. pisum*, highlighting the UPR pathway as a promising target for novel aphid control strategies. These findings advance the development of RNAi-based tools for legume protection while emphasizing the need for further molecular quantification and multi-target optimization.
